# Control of Airborne and Surface Microorganisms in Real Indoor Environments Using an Integrated System of Vaporized Free Chlorine Components and Filtration

**DOI:** 10.3390/microorganisms13092053

**Published:** 2025-09-03

**Authors:** Saki Kawahata, Mayumi Kondo, Atsushi Yamada, Naoya Shimazaki, Makoto Saito, Takayoshi Takano, Tetsuyoshi Yamada, Yoshinobu Shimayama, Shunsuke Matsuoka, Hirokazu Kimura

**Affiliations:** 1 Panasonic Ecology Systems Co., Ltd., Kasugai 486-8522, Japan; kawahata.saki@jp.panasonic.com (S.K.); yamada.atushi@jp.panasonic.com (A.Y.); takano.takayoshi@jp.panasonic.com (T.T.); yamada.tetsuyoshi@jp.panasonic.com (T.Y.); shimayama.yoshinobu@jp.panasonic.com (Y.S.); matsuoka.shunsuke@jp.panasonic.com (S.M.); 2 Department of Clinical Engineering, Faculty of Medical Science and Technology, Gunma Paz University, Takasaki 370-0006, Japan; kondo@paz.ac.jp (M.K.); n-shimazaki@paz.ac.jp (N.S.); ma-saito@paz.ac.jp (M.S.); 3 Advanced Medical Science Research Center/Research Facility Administration Office, Gunma Paz University Research Institute, Takasaki 370-0006, Japan; 4 Department of Health Science, Gunma Paz University Graduate School of Health Science, Takasaki 370-0006, Japan

**Keywords:** infection control, airborne/surface microorganisms, real indoor environment, integrated vaporized free chlorine components and filtration system, HOCl, hypochlorous acid

## Abstract

Airborne and surface-residing microorganisms in indoor environments pose potential risks for infectious disease transmission. To address this issue, we developed a composite device combining a generator of vaporized free chlorine components with a fine particle removal filter. Field tests were conducted in occupied university classrooms to evaluate the device’s efficacy in reducing airborne bacterial loads. Airborne bacteria were sampled under three operational conditions [Electrolyzed (+)/Filter (+), Electrolyzed (−)/Filter (+), and Electrolyzed (−)/Filter (−)]. Significant reductions in bacterial counts were observed in the Electrolyzed (+)/Filter (+) condition, with a residual rate of 14.5% after 2.25 h (*p* = 0.00001). Additionally, surface contact tests demonstrated that vaporized free chlorine components, primarily consisting of hypochlorous acid (HOCl), reduced viable counts of *E. coli*, *P. aeruginosa*, and *S. aureus* by 59.0–99.7% even at a distance of 8.0 m. The concentrations of vaporized free chlorine components during operation remained within safe exposure limits (0–19 ppb), consistent with the effective range reported in prior literature (10–50 ppb). Computational fluid dynamics simulations confirmed the diffusion of vaporized free chlorine components throughout the room, including distant sampling points. These findings suggest the combined use of a vaporized free chlorine generator and a particulate filter effectively reduces microbial contamination in indoor environments, providing a promising approach for infection control in residential and public settings.

## 1. Introduction

Various microorganisms may be suspended in indoor residential environments, posing potential risks for the transmission of infectious diseases [1]. In addition, surfaces of commonly used objects can act as reservoirs for pathogenic organisms, thereby facilitating both direct and indirect contact transmission [2]. These risks are of concern not only in communal living spaces among healthy individuals but also in healthcare settings, such as hospitals and long-term care facilities, where outbreaks can have serious consequences [3]. Therefore, controlling pathogens in residential environments is critical for the prevention of infectious diseases [4]. Recent reports from the World Health Organization (WHO, Geneva, Switzerland) and the Centers for Disease Control and Prevention (CDC) have also highlighted the global health risks posed by airborne and surface-associated microorganisms in indoor environments [5].

To mitigate airborne microorganisms, a variety of devices equipped with fine particle removal filters have been developed [6]. However, such devices are ineffective against microorganisms residing on surfaces. Although liquid disinfectants can reduce microbial loads, only a limited number exhibit broad-spectrum efficacy against diverse pathogens [7]. Furthermore, commonly used disinfectants such as sodium hypochlorite and formalin, while effective against many microorganisms, are limited in applicability due to their high human toxicity, potential release of hazardous gases, and corrosive effects on materials. By contrast, hypochlorous acid-containing gas has been shown to inactivate a wide range of microorganisms, including bacteria and fungi, at concentrations considered safe for human exposure (<0.5 ppm) [8,9,10,11,12,13,14,15,16,17]. Similarly, chlorine dioxide gas has demonstrated inactivation of airborne microorganisms at low concentrations [18].

Based on these considerations, we developed a novel composite device combining a generator of vaporized free chlorine components with a fine particle removal filter. Field tests were conducted in occupied university classrooms to evaluate its efficacy in reducing airborne bacterial loads in an actual environment. Additionally, in an unoccupied laboratory, polystyrene surfaces were inoculated with representative bacterial strains—*Escherichia coli* (*E. coli*), *Pseudomonas aeruginosa* (*P. aeruginosa*), and *Staphylococcus aureus* (*S. aureus*)—to evaluate the device’s ability to inactivate the surface-associated microorganisms.

## 2. Materials and Methods

### 2.1. Integrated System of Vaporized Free Chlorine Components and Filtration

**Figure 1:**
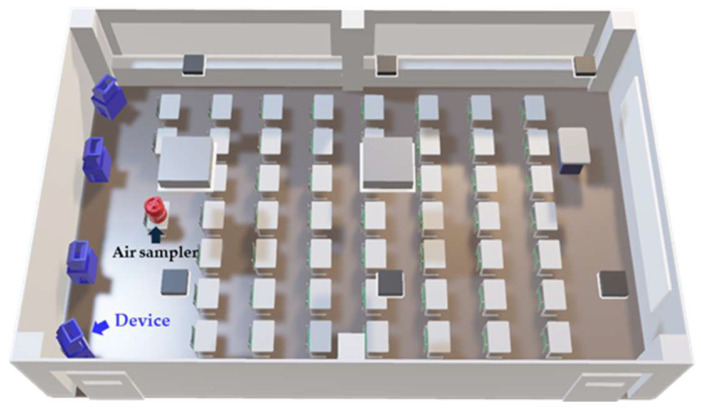
Schematic Overview of the Classroom Used for Airborne Bacteria Testing at the University. Student desks were arranged in rows at the center of the room. Four devices used in the experiment, indicated as the blue structures in the figure, were placed at the rear of the classroom. An air sampler, indicated as a red structure in the figure, was positioned on the student desk at the center of the last row to collect airborne bacterial samples.

This study was conducted in two classrooms routinely used for lectures at Gunma Paz University. No microorganisms were artificially cultured or aerosolized for this study; instead, airborne bacteria were naturally generated by students during lectures, and the measured concentrations reflected human-activity–derived ambient bioaerosols. Both classrooms were nearly identical in terms of size, air conditioning, ventilation capacity, and the desk and chair layout. A schematic overview of the classroom used for airborne bacteria testing at the University is shown in Figure 1. The test device integrated a particulate filtration unit with an evaporative humidification system. The particulate filter consisted of pleated filter media capable of capturing 99.97% or more of particles ≥ 0.3 µm, secured in a frame. The humidification system generated humidified air by forcing airflow through a rotating fiber filter impregnated with electrolyzed water, which was produced by electrolyzing saline solution [19]. The main active component, hypochlorous acid, was volatilized together with water vapor and dispersed as the vaporized free chlorine components. Electrolyzed water was generated by dissolving sodium chloride tablets in tap water and electrolyzing the solution inside the device. For the test, devices equipped with electrolysis electrodes were defined as Electrolyzed (+) and operated electrolyzed water containing approximately 100 mg/L of effective chlorine, which was impregnated into the rotating fiber filter and ventilated at a flow rate of 5.6 m^3^/min. The chosen concentration (<100 mg/L) was based on optimization studies and occupational safety guidelines, ensuring sufficient vaporized free chlorine components while maintaining safety [20]. Devices without electrolysis electrodes using non-electrolyzed water under the same conditions were defined as Electrolyzed (−). The effective chlorine concentration was measured using the H. Chlorine Meter (Kasahara Rika Kogyo Co., Ltd., Kuki, Japan). Devices were further categorized based on the presence of a particulate filter: those equipped with the filter were designated as Filter (+), and those without as Filter (−). Airborne bacteria were sampled over three weeks under three conditions: Electrolyzed (+)/Filter (+), Electrolyzed (−)/Filter (+), or Electrolyzed (−)/Filter (−), each on the same weekday. Preliminary examinations were conducted to determine appropriate operating conditions for vaporized free chlorine components. Although the occupational exposure limit for chlorine gas is 500 ppb, no international standards have been established for vaporized free chlorine components. Considering that medical-grade air sterilization units typically release vaporized free chlorine components below 0.1 ppm (100 ppb), the study was designed to remain within this range. Continuous monitoring demonstrated that the use of four devices in an occupied room (322 m^3^) and two devices in unoccupied room (260 m^3^) maintained vaporized free chlorine components concentrations at ≤17 ppb and ≤19 ppb, respectively. These conditions were therefore adopted for the subsequent airborne and surface bacterial contact experiments.

### 2.2. Environmental Conditions of the Examination Lecture Rooms

The classrooms used for the experiments had a volume of 322 m^3^ (width 12.5 m, depth 9.2 m, height 2.8 m). Each room was equipped with two air conditioning vents and three additional ventilation outlets, providing a total ventilation capacity of 200 m^3^/h, resulting in an air exchange rate of 1.9 times per hour. Four test devices were installed in each classroom and operated continuously for 24 h on weekdays. During sampling, the concentrations of vaporized free chlorine components at the device outlets and within the classroom were continuously monitored and converted to chlorine gas concentration every 10 s using a chlorine gas detector (New Cosmos Electric Co., Ltd., Osaka, Japan) [21]. Room temperature and humidity were recorded near the windows using a Thermo Recorder (Sato Shouji Inc., Kawasaki, Japan). Temperature and humidity variations may influence both bacterial survival and the diffusion of vaporized free chlorine components, potentially acting as confounding factors. Previous studies have shown that higher humidity enhances the diffusion and bactericidal activity of vaporized free chlorine components, while dry conditions may attenuate its efficacy [10].

### 2.3. Airborne Bacteria Sampling Methods

Airborne bacteria were collected using an air sampler (Merck KGaA, Darmstadt, Germany) [1,22]. To cultivate various facultative anaerobic airborne bacteria with high nutritional requirements, nutrient agar supplemented with 5% defibrinated sheep blood (Becton, Dickinson and Company Japan, Tokyo, Japan) [1]. For the Electrolyzed (+)/Filter (+) condition, sampling was conducted at 0 h (before the start of class), 2.25 h, and 4.25 h. The number of occupants at each time point was 22–30, 1–2, and 1–2, respectively. For the Electrolyzed (−)/Filter (+) condition, sampling was conducted at 0 h, 3.75 h, and 5.75 h, with occupant numbers of 27–33, 2–7, and 1–2, respectively. For the Electrolyzed (−)/Filter (−) condition, sampling occurred at 0 h, 2.25 h, and 4.25 h, with 3–24, 1–22, and 1–7 occupants, respectively. Differences in sampling times reflected lecture schedules and occupancy patterns, which affected bacterial generation. Collected samples were incubated at 35 °C for 48 h, after which the number of bacterial colonies was counted [1]. Selected colonies were further analyzed by Gram staining using the Vermy M Staining Kit (MUTO PURE CHEMICALS Co., Ltd., Tokyo, Japan), and species identification was performed using an electron microscope (Nikon Co., Ltd., Tokyo, Japan).

### 2.4. Experimental Conditions for the Contact-Based Bacterial Inactivation Test with Vaporized Free Chlorine Components

**Figure 2:**
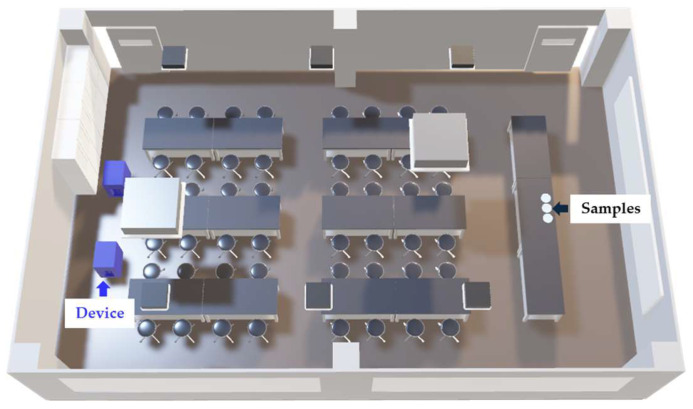
Schematic Overview of the Laboratory Used for Contact Test. The contact test was conducted in a practical training room at the University. Benches and chairs were arranged in the central area of the room. Two experimental devices indicated as the blue structures in the figure were positioned side by side at the rear of the room. The bacterial suspension was applied to dishes, allowed to dry, and subsequently used in the experiments. The dishes were placed on the instructor’s desk at the front of the room, which was 0.8 m high. The distance between the culture dish and the experimental devices was approximately 8.0 m.

The surface bacteria test was conducted in a practical training room at Gunma Paz University. An overview of the contact test is shown in Figure 2. The device used had the same specifications as that employed in the airborne bacteria tests, being equipped with electrolysis electrodes and operated at 5.6 m^3^/min with an effective chlorine concentration of approximately 30 mg/L. In the Electrolyzed (+) group, samples were placed in a room where the device was operated. In the Electrolyzed (−) group, samples were placed inside a W1000 Biological Safety Cabinet (Hitachi Industrial Equipment Systems Co., Ltd., Tokyo, Japan) with its shutter closed. The training room used had a volume of 260 m^3^ (width 10 m, depth 9.3 m, height 2.8 m), with two air conditioning vents and three ventilation inlets, providing a ventilation capacity of 103 m^3^/h, resulting in an air exchange rate of 1.2 times per hour. The room was maintained at 21–27 °C and 40–85% relative humidity using an air conditioner and a steam humidifier (Three-up Co., Ltd., Osaka, Japan). Tests were conducted in an unoccupied environment with two devices installed on one side of the room operating continuously for 24 h. The concentrations of vaporized free chlorine components at the device outlets and sample placement point were continuously detected and converted to chlorine gas concentration every 10 s using a chlorine gas detector (New Cosmos Electric Co., Ltd., Osaka, Japan). Temperature and humidity at the sample sites were recorded every 60 s using a Thermo Recorder (T&D Corporation, Matsumoto, Japan).

### 2.5. Inactivation Test for Bacteria

For the inactivation tests, three bacterial strains were used: *Escherichia coli* (*E. coli*, ATCC 25922 strain), *Pseudomonas aeruginosa* (*P. aeruginosa*, ATCC 27853 strain), and *Staphylococcus aureus* (*S. aureus*, ATCC 29213 strain). Each bacterium was cultured overnight at 37 °C in nutrient broth (KYOKUTO PHARMACEUTICAL INDUSTRIAL Co., Ltd., Tokyo, Japan). On the day of the experiment, each bacterial suspension was adjusted to a turbidity equivalent to McFarland standard 3–4. The *S. aureus* suspension, which had a higher bacterial concentration, was further diluted tenfold with sterile saline. Then, 50 µL aliquots of each bacterial suspension were dropped onto Culture dishes (VTC-D100, AS ONE CORPORATION, Osaka, Japan), air-dried, and placed 8.0 m away from the devices at a height of 0.8 m above the floor. After 24 h, samples were collected (Figure 2). Samples were resuspended in 50 µL of sterile saline, collected using the Dry Transport System (25-806 1PR BT, Sugiyama-Gen Co., Ltd., Tokyo, Japan), diluted, and plated onto Petrifilm. For bacterial counts, SEC plates (Neogen Japan Corporation, Yokohama, Japan) ware used for *E. coli*, AC plates (Neogen Japan Corporation, Yokohama, Japan) for *P. aeruginosa*, and STX plates (Neogen Japan Corporation, Yokohama, Japan) for *S. aureus*. After 24 h of incubation at 37 °C, colony counts were determined.

### 2.6. Simulation of the Concentration of Vaporized Free Chlorine Components

To evaluate the diffusion behavior of the vaporized free chlorine components released from the device to bacteria-exposed dishes located 8.0 m away, a simulation of time-dependent vaporized free chlorine components concentration was conducted using proprietary technology developed by our company (Panasonic Ecology Systems Co., Ltd., Kasugai, Japan). The simulation was performed with STREAM V2022.1, a three-dimensional structured mesh-based computational fluid dynamics (CFD) system. The analysis incorporated factors such as airflow volume, direction, and vaporized free chlorine components concentration at the outlet, along with ventilation and air conditioning conditions (direction and volume). Additional effects, including wall adsorption, removal by ventilation/air conditioning, and self-decomposition, were considered in the transient simulation. Results were visualized as temporal changes in vaporized free chlorine concentration using color mapping over a period of 0 to 1800 s, with 1800 s representing the point at which the concentration stabilized. The CFD simulation was primarily governed by the continuity and Navier–Stokes equations, with the standard k–ε model adopted as the turbulence model. The mesh resolution was set to a reference size of 80 mm, with a finer rectangular parallelepiped mesh applied to regions where significant changes in vaporized free chlorine concentration occurred, such as near walls or around the device. For the boundary conditions, a flow boundary (for the modeled device, ventilation/air-conditioning outlets, airflow volume, vaporized free chlorine concentration, etc., under the same conditions as in this test) and a wall boundary (no-slip condition in which the flow slows due to wall resistance) were applied. In addition, the main factors contributing to vaporized free chlorine component loss, including wall adsorption, removal by ventilation/air-conditioning, and self-decomposition, were separately incorporated based on measured values [23].

### 2.7. Statistical Analyses

Statistical analyses were performed using EZR Ver. 4.3.1 (Easy R, developed by Dr. Kanda at Jichi Medical University, Shimotsuke, Japan), with multiple comparisons conducted by Tukey’s method or Mann–Whitney U test. The level of statistical significance was set at *p* < 0.05.

## 3. Results

### 3.1. Airborne Bacteria Tests

**Figure 3:**
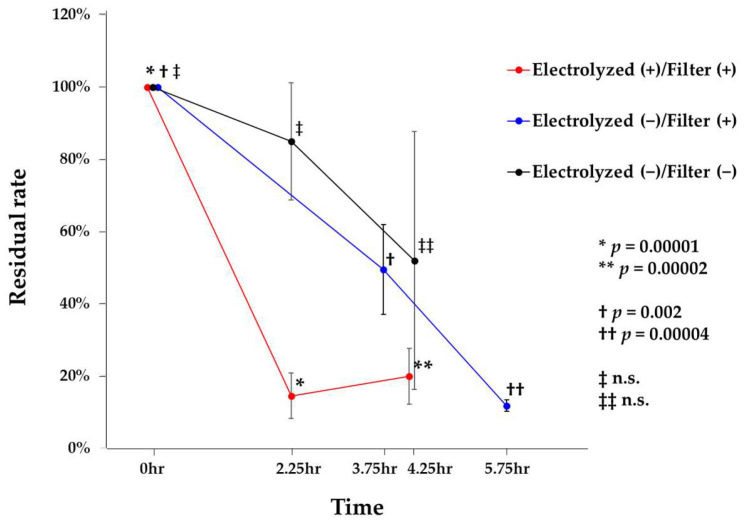
Residual rates of airborne bacteria under three experimental conditions. Residual rates of airborne bacteria were evaluated under three conditions: Electrolyzed (+)/Filter (+), Electrolyzed (−)/Filter (+), and Electrolyzed (−)/Filter (−). Baseline values at 0 h were set to 100% for each sampling session and mean residual rates at subsequent time points were plotted (*n* = 3). The *y*-axis represents the residual rate (%), and the *x*-axis represents the sampling time. Error bars represent the standard deviation (SD). Statistical comparisons were conducted using Tukey’s test. Under the Electrolyzed (+)/Filter (+) condition, a significant reduction was observed between 0 h and 2.25 h (*p* = 0.00001). Under the Electrolyzed (−)/Filter (+) condition, a significant reduction was also observed between 0 h and 3.75 h (*p* = 0.002). Other significant differences are indicated in the figure. n.s., not significant.

Residual rates of airborne bacteria at each sampling time point and temporal changes are shown in Figure 3. When the bacterial count at 0 h was set to 100%, the residual rate under the Electrolyzed (+)/Filter (+) condition significantly decreased to 14.5% at 2.25 h (*p* = 0.00001). During this period, the concentration of vaporized free chlorine components in the air ranged from 4 to 17 ppb. Under the Electrolyzed (−)/Filter (+) condition, the residual rate decreased to 49.4% at 3.75 h, representing a significant reduction compared to 0 h (*p* = 0.002). In contrast, under the Electrolyzed (−)/Filter (−) condition, bacterial counts were measured at 2.25 and 4.25 h; however, no significant reductions were observed. Environmental conditions in the classroom during the test period ranged from 17.5 to 25.7 °C for temperature and 35.7–79.3% for relative humidity. Gram staining of airborne bacteria collected using the air sampler revealed the presence of Gram-positive cocci, Gram-positive bacilli, Gram-negative cocci, yeast-like fungi, and filamentous fungi. These findings indicate the presence of a diverse range of airborne microorganisms in the occupied classroom. Collectively, these results suggest that the combined use of the filter unit installed in the tested device and vaporized free chlorine components effectively reduces airborne bacterial concentrations in occupied indoor environments. Furthermore, the combination of vaporized free chlorine components—primarily composed of hypochlorous acid—with the dust collection filter exhibited superior efficacy in removing airborne bacteria compared to the use of the filter alone.

### 3.2. Contact Tests for Bactericidal Inactivation

Table 1 shows the number of bacteria detected under Electrolyzed (−) and Electrolyzed (+) conditions for the three tested bacterial species in the contact assay, along with the reduction rates under Electrolyzed (+) relative to Electrolyzed (−) and the corresponding *p*-values. Regarding *E. coli*, the bacterial count under Electrolyzed (+) conditions (6.9 × 10^2^ CFU/mL) was significantly reduced by 94.7% compared with that under Electrolyzed (−) conditions (1.3 × 10^4^ CFU/mL) (*n* = 4, *p* = 0.0286). At this time, the concentration of vaporized free chlorine components was 10.8 ppb on average (range: 7–17 ppb) at the air outlet and 3.2 ppb on average (range: 2–4 ppb) at the sample location. For *P. aeruginosa*, the bacterial count under Electrolyzed (+) conditions (5.5 × 10^2^ CFU/mL) was significantly reduced by 99.7% compared to that under Electrolyzed (−) conditions (1.7 × 10^5^ CFU/mL) (*n* = 4, *p* = 0.0286). The concentration of vaporized free chlorine components at this time was 7.2 ppb on average (range: 0–14 ppb) at the air outlet and 0.8 ppb on average (range: 0–2 ppb) at the sample location. For *S. aureus*, the bacterial count under Electrolyzed (+) conditions (3.1 × 10^4^ CFU/mL) was significantly reduced by 59.0% compared with that under Electrolyzed (−) conditions (7.5 × 10^4^ CFU/mL) (*n* = 6, *p* = 0.005). The concentration of vaporized free chlorine components was 12.6 ppb on average (range: 7–19 ppb) at the air outlet and 2.0 ppb on average (range: 0–4 ppb) at the sample location. These results suggest that vaporized free chlorine components, primarily hypochlorous acid, effectively reduce surface-associated *E. coli*, *P. aeruginosa*, and *S. aureus* even at distances of approximately 8.0 m in practical indoor environments.

### 3.3. Simulation Analysis of Vaporized Free Chlorine Components

**Figure 4:**
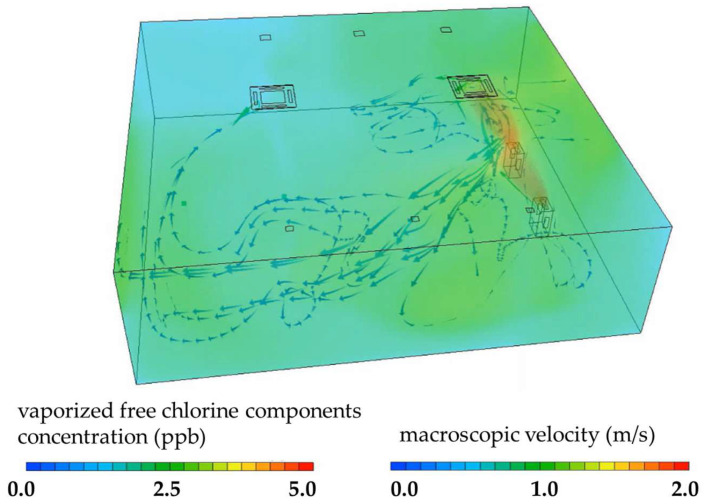
Simulation Analysis of Vaporized Free Chlorine Components. The concentration of vaporized free chlorine components and the simulation results of the humidified airflow emitted from the device at 1800 s after the start of operation are shown. The concentration of vaporized free chlorine components is represented using color mapping. The direction of the humidified airflow emitted from the device is indicated by arrows, and the airflow velocity is visualized using the color of the arrows.

Simulation analyses of the vaporized free chlorine components diffusion and airflow dynamics are presented in Figure 4. After 1800 s of device operation, the simulation showed that the vaporized free chlorine components had filled the practical training room and reached a stable concentration. Color mapping indicated that the vaporized free chlorine components had reached locations approximately 8.0 m away from the device, consistent with the measured vaporized free chlorine components concentrations (0–4 ppb) obtained using a chlorine gas detector.

## 4. Discussion

In the present study, we showed the effectiveness of combining a dust collection filter with vaporized free chlorine components—primarily hypochlorous acid—in reducing airborne bacterial load in an occupied indoor environment. Sampling times varied due to lecture schedules; while this variation may limit comparability, the observed reduction trends remain valid. The convergence of CFU values after approximately 5 h likely reflects multiple factors inherent to occupied indoor spaces, including time-varying occupancy and resuspension, ventilation-driven dilution, and particle deposition. These processes can counteract net removal by filtration over extended periods. Under Electrolyzed (+)/Filter (+) conditions, the residual rate of airborne bacteria significantly decreased compared to control settings, with a notable reduction to 14.5% at 2.25 h (Figure 3). Moreover, contact-based bacterial inactivation tests showed significant reductions in the viable counts of *E. coli*, *P. aeruginosa*, and *S. aureus* under Electrolyzed (+) conditions (Table 1). Reductions ranged from 59.0% to 99.7% across bacterial species and methods. Although the device used in this study was equipped with high-efficiency particulate air filters, the present findings suggest that the removal of airborne microorganisms was substantially influenced by vaporized free chlorine components (Figure 3).

Hypochlorous acid (HOCl) is a potent oxidizing disinfectant with broad-spectrum antimicrobial activity [24]. Thus, the agent is widely used for medical disinfection, food hygiene, and environmental sanitation [7]. It is also produced by some immune cells, including neutrophils, monocytes, and macrophages, as part of the innate immune response, contributing to its high biocompatibility and perceived safety [15,25]. Moreover, vaporized HOCl has garnered increasing attention as a non-contact disinfectant due to its high antimicrobial efficacy and low toxicity [14,15,16,17]. When dispersed as a gas or fine mist into indoor environments, HOCl can inactivate a broad range of airborne microorganisms, including bacteria, viruses, and fungi, without requiring direct surface application. This property makes it particularly advantageous for use in occupied spaces, where continuous or intermittent air disinfection is required. The antimicrobial efficacy of vaporized HOCl may be influenced by several factors, with concentration being one of the most critical determinants [11]. Previous studies suggest that even at low concentrations—typically ranging from 10 to 50 parts per billion (ppb) in the air—vaporized HOCl can exert a significant inactivating effect on airborne microorganisms [11,17]. The classroom’s ventilation rate (1.9 exchanges/hour) may have reduced vaporized free chlorine components residence time, influencing inactivation efficacy. While ventilation diluted vaporized free chlorine components and shortened its residence time, it also enhanced the ecological validity of the results, reflecting real occupied environments. As a result, vaporized free chlorine components, primarily HOCl, at these concentrations effectively inactivated both airborne and surface-associated microorganisms in the present results (Figure 3 and Table 1). These results suggest that our device may contribute to reducing or inactivating various microorganisms in indoor environments, potentially representing the first such observations. Moreover, our simulation showed that the vaporized free chlorine components rapidly filled the practical training room and reached a stable concentration. The results suggest that vaporized free chlorine components, even at low concentrations (around 2 ppb), can effectively reduce airborne bacterial contamination over a distance of approximately 8.0 m, supporting their applicability in real indoor environments such as classrooms.

Next, this study identified a diverse array of airborne microorganisms present in residential indoor settings. Although anaerobic bacteria were not cultured, Gram staining of samples collected via air sampling revealed the presence of Gram-positive cocci, Gram-positive bacilli, Gram-negative cocci, yeast-like fungi, and filamentous fungi. These findings indicate that enclosed, occupied living spaces harbor taxonomically and morphologically diverse airborne microbiota. Given their potential implications for indoor air quality and associations with respiratory infections and allergic responses, the characterization and control of airborne microbial populations represent important considerations for environmental health and public safety [26,27]. Therefore, further studies using our device may be required to facilitate the practical implementation. Although yeast-like and filamentous fungi were detected in airborne samples, the antifungal efficacy of the device was not tested. Future work should evaluate this aspect, as fungi, particularly spore-forming species, may exhibit greater resistance than bacteria, underscoring the need for targeted antifungal evaluations [28].

In this study, we used *S. aureus*, *E. coli*, and *P. aeruginosa* as test organisms in contact-based inactivation experiments using vaporized free chlorine components. These bacterial species were selected due to their clinical and environmental relevance, as well as their frequent use as indicator organisms for evaluating antimicrobial efficacy [29]. *S. aureus* is a Gram-positive coccus commonly found on human skin and mucosal surfaces and is a major contributor to hospital-acquired infections and foodborne contamination [30,31]. *E. coli*, a Gram-negative rod-shaped bacterium, is a typical member of the intestinal microbiota and is widely used as a hygienic indicator organism [32]. *P. aeruginosa*, also a Gram-negative rod, is a non-fermenting bacterium that is ubiquitous in environmental sources and is notable for its high intrinsic resistance to disinfectants and its role as an opportunistic pathogen in immunocompromised individuals [3]. Due to its thick peptidoglycan layer, Gram-positive *S. aureus* exhibits greater resistance to oxidative stress than Gram-negative species. In the present study, vaporized free chlorine components exposure, *S. aureus* exhibited lower inactivation efficiency compared with *P. aeruginosa* and *E. coli* (Table 1). This reduced susceptibility may be attributed to its protective carotenoid staphyloxanthin, which neutralizes reactive chlorine species, and a bacillithiol-based redox system that repairs HOCl-induced protein damage. *S. aureus* requires higher minimal bactericidal concentrations of HOCl than Gram-negative bacteria, reflecting intrinsic resistance [33,34]. Moreover, structural clustering of cocci may also hinder gaseous penetration. Thus, both biochemical defenses and morphological features explain the comparatively lower inactivation efficiency. These organisms differ in morphology, cell wall structure, and disinfectant susceptibility [35], making them suitable model bacteria for comprehensively assessing the bactericidal spectrum of vaporized free chlorine components, primarily HOCl.

Moreover, present simulation data support the hypothesis that vaporized free chlorine components, primarily HOCl, can effectively diffuse throughout indoor environments under realistic operating conditions. The agreement between the simulated diffusion patterns and the empirical vaporized free chlorine components measurements (0–4 ppb) supports the validity of the computational model and suggests that the device is capable of achieving uniform dispersion over distances of at least 8.0 m (Figure 4). Such spatial reach is critical in practical applications, as it indicates that antimicrobial action can be exerted not only near the device but also in distant areas of the room. Furthermore, the ability of the vaporized free chlorine components to maintain a stable concentration over time demonstrates the feasibility of continuous operation for long-term environmental disinfection. These findings underscore the potential utility of combining vaporized free chlorine components generation with fine particle filtration for broad-spectrum infection control in occupied indoor spaces, including educational and healthcare settings. To the best of our knowledge, this is among the first studies to validate the combined use of particulate filtration and vaporized free chlorine components in real occupied environments, highlighting its novelty and practical significance.

Finally, this study has several limitations. First, the experiments were conducted over a short duration and within a specific indoor environment (i.e., a classroom), which may limit the generalizability of the findings to other settings such as hospitals, long-term care facilities, or residential spaces. Long-term impacts on beneficial indoor microbiota and risks of material degradation (e.g., metal corrosion, fabric damage) should be considered for practical applications [36]. Second, microbial identification relied on culture-based methods and Gram staining, without the use of molecular techniques for precise species-level identification or comprehensive microbiome analysis [37]. Anaerobic bacteria were not cultured, and thus the assessment does not account for all microbial risks potentially present in residential environments. Species-level identification was not undertaken because the primary endpoint of the study was device performance under practical classroom conditions rather than microbiological characterization. Nevertheless, limited taxonomic profiling (e.g., culture-based isolates or 16S rRNA sequencing) would provide mechanistic insight into organism-specific removal or inactivation, and will be incorporated into future investigations. Third, the long-term effects of vaporized free chlorine components exposure to indoor environments and human health were not evaluated, and the overall safety profile requires further investigation [38]. Although residual chlorine declined rapidly after shutdown, potential effects of chronic low-level exposure during repeated use remain to be assessed. Next, in this study, the Electrolyzed (−) condition for the contact tests was conducted inside a biological safety cabinet (BSL-2) to exclude vaporized free chlorine components exposure, as no additional training room was available to perform the control under the same schedule as the contact tests. While this approach minimized the risk of airborne or settled bacterial contamination during the 24 h experimental period, we acknowledge that conducting controls under identical environmental conditions would provide more robust and comparable data. Moreover, for the airborne bacteria tests, a no-air-flow control would help to isolate the contribution of filtration alone. Because the objective of this study was to evaluate device performance under real-use conditions with the system operating in an occupied classroom, such a control was not implemented in the present field study. This is acknowledged as a limitation, and a controlled chamber study incorporating a no–air-flow arm is planned for future work. Together, these limitations and uncertainties highlight areas that remain to be elucidated in future investigations.

## 5. Conclusions

In conclusion, the present study demonstrated that combining a high-efficiency dust collection filter with vaporized free chlorine components effectively reduces airborne bacterial contamination in occupied indoor environments. The results showed significant reductions in both airborne and surface-associated viable bacteria under low-concentration vaporized free chlorine component exposure, supporting its potential as a practical, non-contact disinfection strategy. Furthermore, our simulation analyses suggested that vaporized free chlorine components, primarily vaporized HOCl, can disperse uniformly and maintain effective concentrations over substantial distances, thereby extending the antimicrobial effect throughout the indoor space. Although the study was limited by its scope, duration, and reliance on culture-based microbial identification, the findings provide important evidence for the feasibility of using vaporized free chlorine components for continuous environmental disinfection in real-world settings such as classrooms. Future research should investigate the long-term safety of repeated exposure, evaluate efficacy across diverse indoor environments, and incorporate advanced microbial community analyses to better understand the spectrum of inactivation. Together, these efforts will support the development of safer, more effective air and surface disinfection technologies aimed at enhancing indoor environmental health.

## Figures and Tables

**Table 1:** Inactivation of Various Microorganisms through Exposure to the Vaporized Free Chlorine Components.

Bacteria	Electrolyzed (−) (CFU/mL)	Electrolyzed (+) (CFU/mL)	Reduction Rate (%)	*p* Value
*E. coli*	1.3 × 10^4^ ± 5.2 × 10^3^	6.9 × 10^2^ ± 3.0 × 10^2^	94.7 ± 2.3	0.0286
*P. aeruginosa*	1.7 × 10^5^ ± 1.6 × 10^5^	5.5 × 10^2^ ± 6.9 × 10^2^	99.7 ± 0.4	0.0286
*S. aureus*	7.5 × 10^4^ ± 2.4 × 10^4^	3.1 × 10^4^ ± 5.8 × 10^3^	59.0 ± 7.7	0.005

Data are expressed as mean ± standard deviation (SD). Detailed procedures are described in the text.

## Data Availability

The original contributions presented in this study are included in the article. Further inquiries can be directed to the corresponding author.

## References

[1] Matsuki M., Mano Y., Furuya N. (2019). Distribution Survey of Floating Bacteria in General Environments by Air Sampler. Jpn. J. Environ. Infect..

[2] Terayama K., Taneichi F., Honma H., Kawarabayashi T., Yokota M., Aoi Y., Nakata H. (1980). Studies on Bacterial Aerosol Part 5 Analysis of Airborne, Fallen and Floor Bacteria in the Room. Jpn. J. Hyg..

[3] Naru K., Matsunaga N., Noguchi N., Nojo J., Tomizawa T., Ishii T., Namiki U., Yamanaka Y., Kumaki U., Suwa J. (2008). Microbial Surveillance Conducted for Infection Control at a Hospital. Jpn. J. Pharm. Health Care Sci..

[4] Ishimatsu S. (2019). Recent Topics on Microbes in Indoor Environment (4) Microbial Contamination and Countermeasures in Work places. Indoor Environ..

[5] Centers for Disease Control and Prevention (CDC) C. Air|Infection Control. https://www.cdc.gov/infection-control/hcp/environmental-control/air.html.

[6] Imai H., Endo S., Horiuchi Y., Yoshida M., Kaku M. (2024). Experience with HEPA-Filtered Air Purifiers during the COVID-19 Outbreak. Jpn. J. Environ. Infect..

[7] Shiraishi T. (2010). Disinfectant Knowledge You Need to Know. J. Jpn. Soc. Int. Med..

[8] Yoshida S., Muramatsu T., Fukuzaki T. (2016). Volatilization of Hypochlorous Acid from a Solution-Impregnated Fabric Filter in a Forced-Air Vaporizing System. J. Antibact. Antifung. Agents.

[9] Yoshida S., Hayashi T., Ibuka S., Horikiri S., Fukuzaki T. (2019). Effect of Relative Humidity on the Bactericidal Action of Gaseous Hypochlorous Acid against *Staphylococcus aureus* on a Dry Solid Surface. J. Antibact. Antifung. Agents.

[10] Makimura S., Kato R., Yoshida S., Hayashi T., Ibuka S., Horikiri S., Fukuzaki T. (2019). Effect of Chlorine-Consuming Organic Substances on the Bactericidal Action of Gaseous Hypochlorous Acid on a Wet Agar Plate. J. Environ. Control Tech..

[11] Mizuno Y., Ibuka S., Hayashi T., Horikiri S., Fukuzaki T. (2020). Bactericidal Action of Gaseous Hypochlorous Acid against *Staphylococcus epidermidis* in Aerosols in a Confined Space. J. Environ. Control Tech..

[12] Nakamura K., Katou R., Hayashi T., Ishida Y., Horikiri S., Yoshida S., Fukuzaki S. (2021). Volatility and Antimicrobial Actions of Hypochlorous Acid and Monochloramin in a Forced-Air Vaporizing System under Alkaline Conditions. J. Antibact. Antifung. Agents.

[13] Nakamura K., Hotta H., Hayashi T., Ishida Y., Yoshida S., Fukuzaki S. (2021). Indoor Concentration and Bactericidal Action of Gaseous Hypochlorous Acid during the Operation of a Forced-Air Vaporizer Fed with Weakly Alkaline Hypochlorite Solution. J. Environ. Control Tech..

[14] Boecker D., Breves R., Herth F., Zhang Z., Bulitta C. (2022). Safe, Effective, and Cost-Efficient Air Cleaning for Populated Rooms and Entire Buildings Based on the Disinfecting Power of Vaporized Hypochlorous Acid. Int. J. Health Med. Eng..

[15] Boecker D., Zhang Z., Breves R., Herth F., Kramer A., Bulitta C. (2023). Antimicrobial Efficacy, Mode of Action and in vivo Use of Hypochlorous Acid (HOCl) for Prevention or Therapeutic Support of Infections. GMS Hyg. Infect. Control.

[16] Fukuzaki S. (2023). Uses of Gaseous Hypochlorous Acid for Controlling Microorganisms in Indoor Spaces. J. Microorg. Control.

[17] Hayashi T., Mizuno Y., Yoshida S., Fukuzaki T. (2024). Efficacy of Gas-phase Residual Hypochlorous Acid in Disinfecting Bacteria in Aerosols under Low Humidity Conditions. J. Environ. Control Tech..

[18] Ogata N., Sakasegawa M., Miura T., Shibata T., Takigawa Y., Taura K., Taguchi K., Matsubara K., Nakahara K., Kato D. (2016). Inactivation of Airborne Bacteria and Viruses Using Extremely Low Concentrations of Chlorine Dioxide Gas. Pharmacology.

[19] Kato R., Makimura S., Yoshida S., Muramatsu T., Hayashi T., Ibuka S., Fukuzaki T. (2018). Analysis of Stripping Process of Hypochlorous Acid in a Forced-Air Vaporizing System. J. Environ. Control Tech..

[20] Nakato T., Hamatani M., Nikaido M., Morozumi H., Nishiki Y., Tsuchizaki N., Sudo Y., Kikuchi K., Hotta K. (2018). Establishment of JIS B 8701 for Hypochlorous Acid Water producing Apparatus. J. Func. Water.

[21] Yoshida S., Hayashi T., Kato R., Kusakawa T., Fukuzaki T. (2017). Simple Measurement of Gaseous Hypochlorous acid using a Chlorine Gas Detector Equipped with a Controlled Potential Electrolysis Sensor. J. Environ. Control Tech..

[22] Shibuya K. (2003). Determining Airborne Biocontamination. J. Aerosol. Res..

[23] Yi J., Lee J., Fikri M.A., Sang B.-I., Kim H. (2020). Application of Computational Fluid Dynamics in Chlorine-Dynamics Modeling of In-Situ Chlorination Systems for Cooling Systems. Appl. Sci..

[24] Kawaguchi Y., Oie S., Furukawa H. (2016). Efficacy of Complex-Type Chlorin-Based Disinfectant Cleaner against MDRP and MDRA. Jpn. J. Environ. Infect..

[25] Aratani Y. (2006). Role of Myeloperoxidase in the Host Defense against Fungal Infection. Jpn. J. Med. Mycol..

[26] Ueda S., Kuwabara Y. (1980). Air Borne Bacteria and Bacilli in a Kitchen. J. Food Sci. Technol..

[27] Kawakami Y. (2018). Recent Topics on Microbes in Indoor Environments (1) Introduction: Changes in Housing Patterns and Deseases Associated with Problems of Indoor Environmental Microbes. Indoor Environ..

[28] Nakamura S., Kuwahara M., Fukuda K., Yamanaka N., Ishihara M. (2017). Bactericidal Effect of HOCl Aqueous Solution on Microorganisms Such as Viruses and Its Application. J. Natl. Def. Med. Coll..

[29] Miyashita M., Sugawa M., Sone A., Sato K., Tsuruya S., Hirose K., Mori M., Hayashi H., Komoriyama H., Kawashima Y. (2011). Effectiveness of Supplying Enteral Nutrition Formulas through Newly Developed Disposal Containers, the Second Report-The Investigation on Sanitary Conditions. J. Jpn. Dietet. Assoc..

[30] Taguchi F., Saito-Taki T., Okuda S., Aoki M., Matsuzaki T., Tomioka M., Kikuno R., Lee S.M. (1992). Proposal for the Nosocomial Infection Control of Methicilin-Resistant *Staphylococcus aureus* (MRSA). Jpn. J. Bacteriol..

[31] Takahashi T., Onoue Y., Mori M. (1979). Monthly Variation of Bacterial Contamination and Incidence of *Staphylococcus aureus* in a Japanese Lunch “Makunouchi-Bento”. Food Hyg.Saf..

[32] Nakamura H., Ogasawara J., Yasufuku K., Arikawa K., Ohyama M., Abe N., Hase A. (2012). Identification and Comparison of Isolates from Several Coliform Group Detection Agar Medium. Jpn. J. Food. Microbiol..

[33] Clauditz A., Resch A., Wieland K.-P., Peschel A., Gotz F. (2006). Staphyloxanthin Plays a Role in the Fitness of *Staphylococcus aureus* and Its Ability To Cope with Oxidative Stress. Infect. Immun..

[34] Linzner N., Loi V.V., Fritsch V.N., Antelmann H. (2021). Thiol-Based Redox Switches in the Major Pathogen *Staphylococcus aureus*. Biol. Chem..

[35] Kato I. (2019). Microbiological Knowledge Necessary for Understanding and Analyzing Data from JANIS. Jpn. J. Environ. Infect..

[36] Chigusa H., Okawa T., Yokota M., Nikaido M., Matsumura Y., Iwasawa A. (2017). Bactericidal and Corrosive Properties of Hypochlorous Acid Water at Various pH and Available Chlorine Concentrations. J. Antibact. Antifung. Agents.

[37] Hattori M. (2014). Advanced Technologies for the Human Gut Microbiome Analysis. Jpn. J. Clin. Immunol..

[38] Muramatsu T., Kodama K., Yamada T., Yamada A., Fukuzaki S. (2024). Inhalation of Gaseous Hypochlorous Acid and Its Effect on Human Respiratory Epithelial Cells in Laboratory Model Systems. J. Microorg. Control.

